# Deep graph level anomaly detection with contrastive learning

**DOI:** 10.1038/s41598-022-22086-3

**Published:** 2022-11-18

**Authors:** Xuexiong Luo, Jia Wu, Jian Yang, Shan Xue, Hao Peng, Chuan Zhou, Hongyang Chen, Zhao Li, Quan Z. Sheng

**Affiliations:** 1 School of Computing, Macquarie University, Sydney, Australia; 2 School of Computing and Information Technology, University of Wollongong, Wollongong, Australia; 3 Beijing Advanced Innovation Center for Big Data and Brain Computing, Beihang University, Beijing, China; 4 Academy of Mathematics and Systems Science, Chinese Academy of Sciences, Beijing, China; 5 Zhejiang Lab, Hangzhou, China; 6 Hangzhou Yugu Technology, Hangzhou, China

**Keywords:** Computational science, Computer science

## Abstract

Graph level anomaly detection (GLAD) aims to spot anomalous graphs that structure pattern and feature information are different from most normal graphs in a graph set, which is rarely studied by other researchers but has significant application value. For instance, GLAD can be used to distinguish some different characteristic molecules in drug discovery and chemical analysis. However, GLAD mainly faces the following three challenges: (1) learning more comprehensive graph level representations to differ normal graphs and abnormal graphs, (2) designing an effective graph anomaly evaluation paradigm to capture graph anomalies from the local and global graph perspectives, (3) overcoming the number imbalance problem of normal and abnormal graphs. In this paper, we combine graph neural networks and contrastive learning to build an end-to-end GLAD framework for solving the three challenges above. We aim to design a new graph level anomaly evaluation way, which first utilizes the contrastive learning strategy to enhance different level representations of normal graphs from node and graph levels by a graph convolution autoencoder with perturbed graph encoder. Then, we evaluate the error of them with corresponding representations of the generated reconstruction graph to detect anomalous graphs. Extensive experiments on ten real-world datasets from three areas, such as molecular, protein and social network anomaly graphs, show that our model can effectively detect graph level anomaly from the majority and outperform existing advanced methods.

## Introduction

Graph structure which contains node set and edge link set between nodes is commonly used to model different domain data, such as molecular graph, traffic graph and brain structure graph. For example, for a chemical molecule, we can consider atoms as nodes and chemical bonds as edges to construct its graph structure. In recent years, data mining research based on graph information^1,2^ attracts increasing attention because graph structure information can be used to model different application scenarios. Then, the purpose of graph level anomaly detection (GLAD) task is to detect rare graph patterns that differ from the majority of graphs, which can be employed to spot toxic molecules in chemical compounds analysis^3,4^, recognize patient neuroimaging in disease diagnosis^5^, and detect abnormal internet activity graphs^6^.

**Figure 1:**
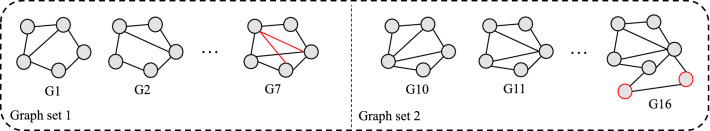
The toy example shows two types of graph level anomaly, where graph set 1 and graph set 2 are two kinds of modeled molecule graph, respectively. For the graph set 1, other molecule graphs are ring structure with three node ring and four node ring, but G7 only has three node ring due to the anomalous links of one node, i.e., local graph anomaly. For the graph set 2, G16 adds two new nodes, which shows different structure patterns with other molecule graphs from the whole graph perspective, i.e., global graph anomaly.

Recently, many graph anomaly detection methods are proposed but they focus on node level anomaly detection which finds abnormal nodes that have obvious difference with other nodes within a single graph. ComGA^7^ analysed multiple anomalies of nodes within a graph, and utilized graph neural networks (GNN) model to capture anomaly-aware node representations containing community structure information. Then, more node level anomaly detection methods^8–10^ are proposed by analyzing the implicit anomaly information and utilizing advanced deep learning techniques. However, how to conduct GLAD receives little attention. The main reason is that GLAD faces huger challenge compared with node level anomaly detection task: (1) in order to observe the difference of anomalous graphs with others in a GLAD framework, this not only needs to consider local graph anomaly pattern, but also needs to capture the difference of global graph structure. As shown in Fig. 1, when we detect some chemical molecules that their molecular graphs represent obvious difference with others in global graph pattern, we need to consider global graph anomaly. However, if we want to spot these similar chemical molecules that has the difference in local structure, we should capture local graph anomaly. Thus, we cannot only consider how to learn better node level representations for local graph anomaly pattern like the node level anomaly detection task, but also need to learn more comprehensive graph level representations, which decides on the performance of abnormal graphs detection. However, effective graph level representations are still a difficult problem apart from the traditional graph pooling methods^11^, such as max pooling or global pooling. (2) Unlike that the node level anomaly detection task utilizes node representation reconstruction error to detect anomalous nodes^7,12,13^ or the two-stage graph anomaly detection methods detect anomalous graphs based on graph level representations^14^ , we need to design a reliable and oriented GLAD evaluation method by the end-to-end framework to judge anomalous graphs. (3) In real-world application scenarios, the number of anomalous graphs is few. Besides, anomalous graph patterns are unseen. This enables the designed GLAD model to be trained by an unsupervised way and meet the imbalance of anomalous graph class, which is different from graph classification task that is supervised or semi-supervised learning ways and needs graph label information.

To tackle the mentioned challenges, in this work, a deep Graph Level Anomaly Detection with Contrastive learning (GLADC) framework is proposed, which aims to design a new graph level anomaly evaluation paradigm. Specifically, we use graph contrastive learning method to enhance node level and graph level representations of normal graphs and measure anomalous graphs by the latent graph representation errors from real input graphs and generated reconstruction graphs. The framework is organized by the following three modules: (1) dual-graph encoder module consists of graph convolution autoencoder and perturbed graph encoder, which is used to learn node level representation by encoding and decoding the graph, and learns graph level representation by graph contrastive learning strategy. (2) Graph encoder module mainly learns latent graph representations in node level and graph level by encoding generated reconstruction graph, which is adapted to the inference of anomalous graph representations. (3) Graph anomaly detection module detects anomalous graphs by evaluating the error of generative graph representations and input graph representations instead of relying on the reconstruction error for anomaly detection. To train the GLADC framework by an end-to-end way and avoid the imbalance of graph class. we first use normal samples to train the former two modules to learn normal graph representation distributions. Then, when we input test samples containing normal and abnormal graphs, graph anomaly detection module will generate bigger error for abnormal graphs because GLADC cannot effectively learn anomalous graph representations.

We notice that contrastive learning method has been used to graph anomaly detection^8,10^, but most of them focus on node level contrastive learning and only aim to detect node level anomaly. Furthermore, GLocalKD^15^ proposed a joint random distillation framework for graph and node representations to detect anomalous graphs, but it cannot learn more effective graph representations for GLAD. To summarize, the highlighted contributions of GLADC are three-fold:

- We achieve an end-to-end GLADC framework to detect anomalous graphs from local and global graph anomalies.
- We design a new graph level anomaly evaluation paradigm, which first uses graph contrastive learning method to capture node level and graph level representations of normal graphs by a dual-graph encoder module, and then measures anomalous graphs by the error score of generated reconstruction graph representations and input real graph representations.
- We perform extensive experiments on different types of real-world datasets including molecular, protein and social network anomaly graphs to verify the effectiveness of GLADC compared with baselines.

## Related works

### Graph anomaly detection

Graph anomaly detection draws growing interest in recent years. The previous methods^16–20^ mainly designed shallow model to detect anomalous nodes by measuring graph structure and node attributes. Due to the strong graph learning ability of GNN^21^, more graph anomaly detection methods^7,8,10,22–24^ utilized the GNN as backbone and introduced more advanced deep learning models to analyze anomalous nodes. For instance, ANEMONE^22^ considered different graph views information and constructed path level and context level contrastive learning to capture anomalous nodes. Meta-GDN^24^ conducted few-shot network anomaly detection by using few labeled samples for meta-learning algorithm. CoLA^10^ constructed contrastive instance pair from target node and local subgraph to abnormal nodes detection. However, how to realize anomalous graphs detection in a big graph set is rarely studied. Most of traditional GLAD methods^14,25^ applied the two-stage strategy: they first learned graph vector representations by graph kernel methods, e.g., Weisfeiler-Leman kernel^26^ and propagation kernel^27^ or graph embedding learning methods, e.g., Graph2Vec^28^ and FGSD^29^. Second, based on the above graph representations, they then utilized the shallow anomaly detectors to detect abnormal graphs, such as isolation forest (IF)^30^, local outlier factor (LOF)^31^, and one-class support vector machine (OCSVM)^32^. Alternatively, the latest GLAD method GLocalKD^15^ utilized node and graph representations distillation to capture graph level anomaly, but graph level representation is only learned by the simple pooling way, which cannot capture comprehensive graph structure information and limits the performance of GLAD.

### Graph contrastive learning

Contrastive learning^33–35^ utilizes the mutual information maximization mechanism which maximizes instances with similar semantic information to get rich feature representations and is widely used to learn graph representation in recent works. For example, DGI^36^ first introduced contrastive learning into graph representation learning method, and it learned effective graph data representation by local mutual information maximization. GMI^37^ built contrast instances from the perspectives of edge and node to learn node representation. In order to construct effective instance pairs for contrastive learning, GCA^38^ generated different contrast views by an adaptive graph data augmentation way. GraphCL^39^ designed four types of augmentations to generate graph pairs for contrastive learning. MoCL^40^ designed molecular graph augmentations with domain knowledge to preserve related semantic information. But these methods heavily rely on data augmentation way to build contrastive graphs, this easily introduces graph data noise and adds more computer cost, which is not advantageous for GLAD. Thus, the proposed GLADC aims to design a simple and effective graph contrastive learning paradigm for GLAD without graph data augmentation. Furthermore, graph level anomaly detection task is obviously different from other graph analysis task, such as graph representation learning or graph classification tasks. GLAD not only needs fuse graph representation learning method and anomaly detection technique, but also is employed more complex and unknown graph level anomaly detection analysis, such as drug discovery research.

## Problem formulation

Let a graph set ${\mathscr{G}}=\left\{ G_{1},G_{1},\ldots ,G_{m}\right\}$ with normal and abnormal graphs, each graph in ${\mathscr{G}}$ is noted by $G=(\nu _{G},\varepsilon _{G})$, where $\nu _{G}=\left\{ \nu _{1},\nu _{2},\cdots ,\nu _{n}\right\}$ is the number of nodes in the graph and $\varepsilon _{G}=\left\{ e_{1},e_{2},\ldots ,e_{s}\right\}$ is the number of edges. The topology information of each graph is denoted as an adjacency matrix ${\varvec{A}}\in {\mathbb{R}}^{n\times n}$, where ${\varvec{A}}_{i,j}=1$ if $(\nu _{i} ,\nu _{j})\in \varepsilon _{G}$; otherwise, ${\varvec{A}}_{i,j}=0$. If the graph *G* is an attributed graph, nodes are associated with their own attribute features, where the feature matrix of nodes as ${\varvec{X}} \in {\mathbb{R}}^{n\times d}$.

Given a graph set ${\mathscr{G}}$ containing *m* graphs, the proposed model GLADC aims to recognize abnormal graphs that significantly have difference with the majority of normal graphs from the local and global graph anomaly perspectives.

## Methods

**Figure 2:**
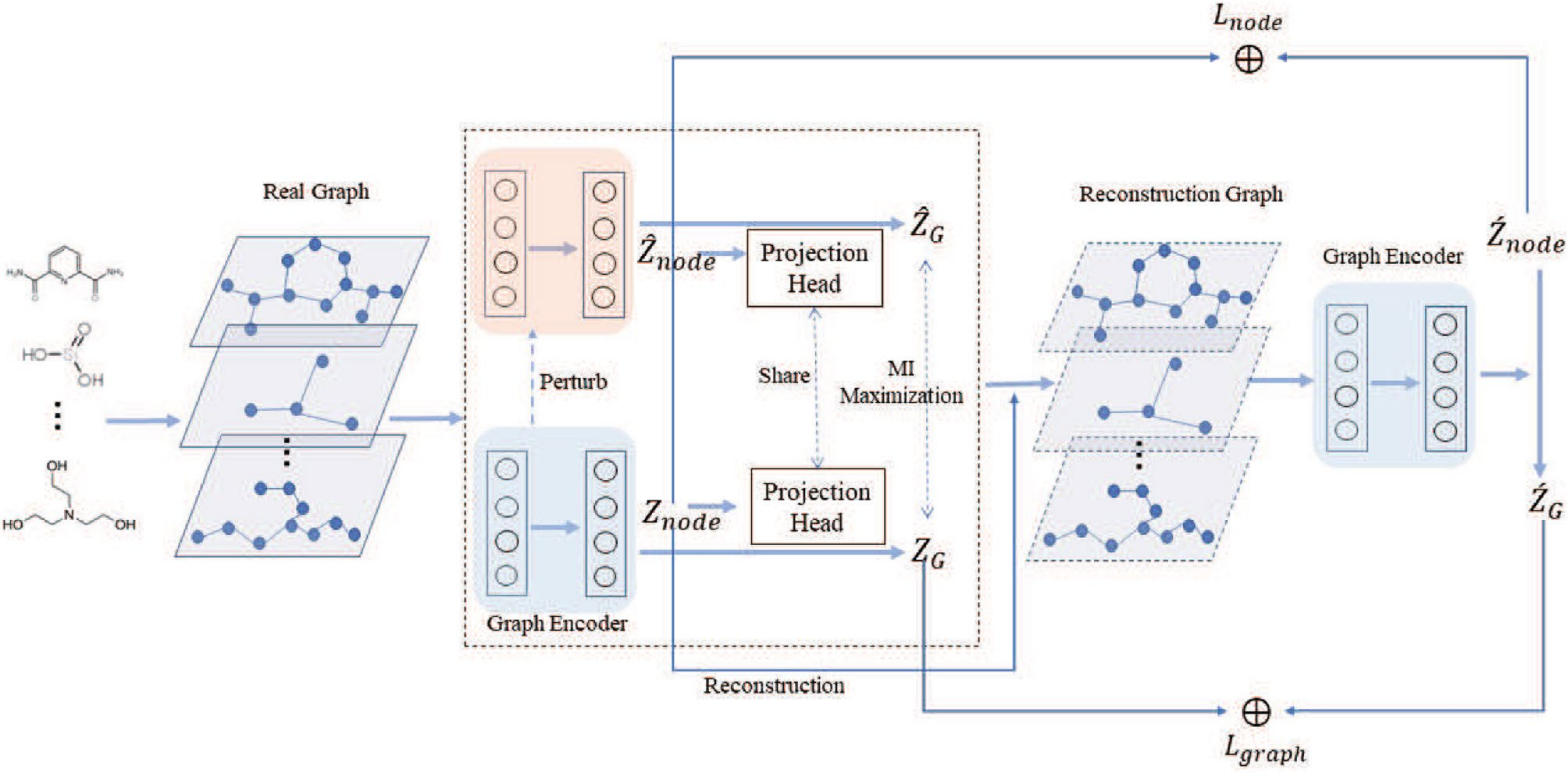
The model architecture of GLADC is constructed by the following three modules. For dual-graph encoder module, we first use a graph convolution autoencoder to learn node level representation as ${\mathbf{Z}}_{node}$ by encoding and decoding the input real graph. Then, we introduce a disturbed graph encoder to get another node level representation as ${\hat{\mathbf{Z}}}_{node}$. We design a contrastive learning paradigm to enhance graph level representation based on initial graph level representations ${\hat{\mathbf{Z}}}_{G}$ and ${\mathbf{Z}}_{G}$ learned by shared projection head. For graph encoder module, we use a shared graph encoder to encode the reconstruction graph to get latent node level and graph level representations as ${\acute{\mathbf{Z}}}_{node}$ and ${\acute{\mathbf{Z}}}_{G}$ respectively. For graph anomaly detection module, we first train GLADC model in normal graphs, and test model by test dataset containing normal and abnormal graphs. Then, anomalous graphs are recognized according to the error score of corresponding graph representations from the input graph and the reconstruction graph.

This section shows how to build deep graph level anomaly detection with contrastive learning framework and how to train the model by the end-to-end way to detect anomalous graphs. The proposed GLADC model is illustrated in Fig. 2.

### Dual-graph encoder module

To detect anomalous graphs for different types of graph anomaly set, we need to consider graph anomaly from the perspectives of local and global graph anomalies. In this work, we use node level and graph level representation errors to realize two types of graph anomaly detection, respectively. Because node level representation more integrates node neighbors’ information and graph level representation pays more attention to the whole graph information. Thus, this enables to learn higher quality graph representations in node level and graph level for a GLAD framework. We first utilize the graph autoencoder to encode and decode graphs and build reconstruction error loss to optimize node level representation. But how to learn an effective graph level representation in the GLAD framework is difficult. Because some traditional graph level representation methods based on graph pooling way cannot capture comprehensive global structure information. Another advanced methods^39,41^ based on graph data augmentation way artificially destroy original graph structure, such as node dropping, edge perturbation and attribute masking, to construct graph contrastive learning framework, which easily increases graph information noise in graph data preprocessing. Besides, the complex process is not practical in large graph anomaly datasets. Inspired by the SimGARCE model^42^ which built graph contrastive instances pair without data augmentation, we design a dual-graph encoder module to learn node level representation and enhance graph level representation. Firstly, we utilize the simple GCN^43^ layer which learns node embedding representation by aggregating node neighbors’ information as the graph encoder $f(\cdot ;{\varvec{\theta }})$. Specifically, the *l*-th layer $f_{l}(\cdot ;{\varvec{\theta }})$ as follows:

1 $$\begin{aligned} f_{l}=\phi \left( {\tilde{\mathbf{D}}}^{-\frac{1}{2}}{\tilde{\mathbf{A}}}{\tilde{\mathbf{D}}}^{-\frac{1}{2}}{\mathbf{Z}}_{l-1}{\varvec{\theta }}_{l-1}\right) , \end{aligned}$$

where ${\tilde{\mathbf{A}}}={\mathbf{A}}+{\mathbf{I}}$ and ${\mathbf{I}}$ denotes the identity matrix for the graph *G*. ${\tilde{\mathbf{D}}}_{ii}=\sum _{j}{\tilde{\mathbf{A}}}_{ij}$ and ${\tilde{\mathbf{D}}}_{ii}$ is the corresponding degree matrix, and ${\varvec{\theta }}$ is the learnable weight matrix of the corresponding GCN encoding layer.

At the same time, we use a random Guassian noise to disturb graph encoder as $f(\cdot ;\acute{\theta })$, where $\acute{\theta _{l}}$ in *l*-th layer as follows:

2 $$\begin{aligned} {\acute{\varvec{\theta }} _{l}}={\varvec{\theta }} _{l}+\eta \cdot \Delta {\varvec{\theta }} _{l} ;\Delta {\varvec{\theta }} _{l}\sim N\left( 0,\sigma _{l}^{2}\right) , \end{aligned}$$

where the coefficient $\eta$ is used to control the size of given perturbation. $\Delta {\varvec{\theta }} _{l}$ denotes the perturbation part which is obtained from Gaussian distribution that mean is 0 and variance is $\sigma _{l}^{2}$.

Then, we use graph max pooling way which computes the max value of related dimensions in all node representations to obtain two graph level representations as ${\hat{\mathbf{Z}}}_{G}$ and ${\mathbf{Z}}_{G}$ from two node level representations ${\hat{\mathbf{Z}}}_{node}$ and ${\mathbf{Z}}_{node}$ learned by $f(\cdot ;\acute{\theta })$ and $f(\cdot ;\theta )$. But the learned node level and graph level representations do not satisfy the request of high-quality graph representations. To get effective node level representation, we use two graph decoders to reconstruct node level representation from graph topology information and node attributes, respectively. The graph structure decoder as follows:

3 $$\begin{aligned} {\hat{\mathbf{A}}}=sigmoid\left( {\mathbf{Z}}_{node}{\mathbf{Z}}_{node}^{T}\right) . \end{aligned}$$

It is noted that we conduct inner product operation between two node representations to evaluate the possibility of existing a link:

4 $$\begin{aligned} p\left( {\hat{\mathbf{A}}}_{ij}=1|{\mathbf{z}}_{node,i},{\mathbf{z}}_{node,j}\right) =sigmoid\left( {\mathbf{z}}_{node,i},{\mathbf{z}}_{node,j}^{T}\right) . \end{aligned}$$

To reconstruct node attributes, we use the GCN layer to achieve the attribute decoder of module:

5 $$\begin{aligned} {\hat{\mathbf{X}}}=\phi \left( {\tilde{\mathbf{D}}}^{-\frac{1}{2}}{\tilde{\mathbf{A}}}{\tilde{\mathbf{D}}}^{-\frac{1}{2}}{\mathbf{Z}}_{node}{\varvec{\theta }}_{d}\right) . \end{aligned}$$

Thus, the reconstruction loss of graph convolutional autoencoder combining structure reconstruction and attribute reconstruction is formulated as:

6 $$\begin{aligned} L_{1}= \left\| {\mathbf{A}}-{\hat{\mathbf{A}}} \right\| _{F}^{2}+\left\| {\mathbf{X}}-{\hat{\mathbf{X}}} \right\| _{F}^{2}. \end{aligned}$$

Specifically, we use generative graph autoencoder to learn node-level representation, which is effective to GLAD task. Because anomalous graphs mainly show the error of structure information and node attributes between graphs. This learning way which reconstructs node feature representation from graph structure and node attributes can highlight the difference of node representation between graphs. But this is not directly employed to GLAD. Because our goal aims to detect anomalous graphs within graph set instead of anomalous nodes within a graph.

To capture more comprehensive graph level representation, we utilize two graph level representations ${\hat{\mathbf{Z}}}_{G}$ and ${\mathbf{Z}}_{G}$ to build a graph contrastive learning paradigm, which avoids graph data noise introduced by graph augmentation and preserves graph structure semantic by the perturbed graph encoder. We first use two-layer perceptron (MLP) as projection head to map two graph level representations into the latent space which can enrich graph information representation as ${\hat{\mathbf{Z}}}_{\acute{G}}$ and ${\mathbf{Z}}_{\acute{G}}$. Then, inspired by the previous works^39,44^, we conduct contrastive instances pair learning based on positive pairs ${\hat{\mathbf{Z}}}_{\acute{G}}$ and ${\mathbf{Z}}_{\acute{G}}$ and negative pairs by the special cross entropy loss. The contrastive learning loss in the *i*-th graph as follows:

7 $$\begin{aligned} L_{2}=-log\frac{exp\left( sim\left( {\hat{\mathbf{Z}}}_{\acute{G}i},{\mathbf{Z}}_{\acute{G}i}\right) /\tau \right) }{\sum _{\acute{i}=1,\acute{i}\ne i}^{N}exp\left( sim\left( {\mathbf{Z}}_{{\acute{G}}i},{\mathbf{Z}}_{{\acute{G}}{\acute{i}}}\right) /\tau \right) }, \end{aligned}$$

where $sim({\hat{\mathbf{Z}}}_{\acute{G}},{\mathbf{Z}}_{\acute{G}})=\frac{{\mathbf{Z}}_{\acute{G}}^{\top }{\hat{\mathbf{Z}}}_{\acute{G}}}{\left\| {\hat{\mathbf{Z}}}_{\acute{G}}\right\| \left\| {\mathbf{Z}}_{\acute{G}} \right\| }$ and $\tau$ is the temperature parameter. And $sim(\cdot ,\cdot )$ is used to compute the cosine similarity. For a minibatch of *N* graphs in training process, we can get 2*N* graph representations because we learn two graph level representations for each graph in above process. Here we consider that negative pairs are sampled from $N-1$ representations generated by perturbed graph encoder within the same mini-batch as in^33^.

### Graph encoder module

Most traditional node level anomaly detection methods utilize the reconstruction error to judge anomalous nodes, but this way is not effective for GLAD. The main reason is that GLAD analyzes the difference between graphs instead of nodes. Thus, we utilize a graph encoder to encode the generated reconstruction graph for new graph representations ${\acute{\mathbf{Z}}}_{node}$ and ${\acute{\mathbf{Z}}}_{G}$ in node level and graph level respectively, which aims to inference anomalous graphs from the error of latent graph representations in node level and graph level compared with ${\mathbf{Z}}_{G}$ and ${\mathbf{Z}}_{node}$. It is noted that the graph encoder is shared with the former graph encoder in dual-graph encoder module. Furthermore, we design the error loss from node level and graph level representations to guarantee the stability of measurement strategy as follows:

8 $$\begin{aligned}&L_{node}=\frac{1}{\left| {\mathscr{G}}\right| }\sum _{G\in {\mathscr{G}}}\left( \frac{1}{\left| G\right| }\sum _{v_{i}\in \nu _{G}}\left\| {\mathbf{Z}}_{node,i}-{\acute{\mathbf{Z}}}_{node,i} \right\| ^{2}\right) , \end{aligned}$$

9 $$\begin{aligned}&L_{graph}=\frac{1}{\left| {\mathscr{G}}\right| }\sum _{G\in {\mathscr{G}}}\left\| {\mathbf{Z}}_{G}-{\acute{\mathbf{Z}}}_{G} \right\| ^{2}, \end{aligned}$$

10 $$\begin{aligned}&L_{3}= L_{node}+L_{graph}. \end{aligned}$$

### Graph anomaly detection module

To detect anomalous graphs from the perspectives of local and global graph anomalies, we utilize the error of node level and graph level representations between input real graphs and generated fake graphs to measure anomalous graphs. Specifically, during the GLADC training, we first use normal graphs train the GLADC model to capture normal node level and graph level representation distributions. Then, we input test graphs containing normal and abnormal graphs into GLADC. When the given graph is abnormal graph, the error of node level and graph level representations will become bigger compared with the given normal graph. Because the training process has learned the normal graph pattern and the generated fake graph also is close to the real graph. However, the abnormal graph pattern is not captured in this training process, and the generated fake graph is not similar with real graph, resulting in the bigger error of node level and graph level representations of fake reconstruction graph with corresponding representations of the input real graph. Thus, we denote the error of node level and graph level representations as the anomaly score as follows:

11 $$\begin{aligned} Score_{G}=\frac{1}{\left| G \right| }\sum _{v_{i}\in \nu _{G}}\left\| {\mathbf{Z}}_{node,i}-{\acute{\mathbf{Z}}}_{node,i}\right\| ^{2}+\left\| {\mathbf{Z}}_{G}-{\acute{\mathbf{Z}}}_{G} \right\| ^{2}. \end{aligned}$$

### Loss function

To detect anomalous graphs from local and global graph anomaly perspectives by an end-to-end way, and optimize the proposed GLADC framework, we joint the above loss functions as a unified loss function as follows:

12 $$\begin{aligned} L_{total}=L_{1}+L_{2}+L_{3}. \end{aligned}$$

## Datasets

**Figure 3:**
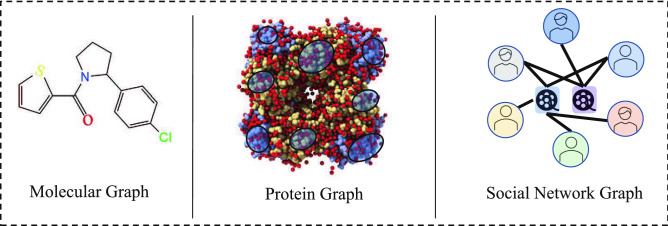
The example of visualization graph for molecular graph, protein graph generated from the public protein file bank^45^ and social network graph.

In this experiments, we select ten public real-world graph anomaly datasets as shown in Table 1 to test the effectiveness of proposed GLADC, where five datasets containing BZR, DHFR, COX2, ENZYMES and AIDS are attributed graphs with node attribute information, and other datasets are plain graphs. For plain graph datasets, the node feature information is used from the degree information of each node. Furthermore, it is worth noting that the reported anomalous ratio of each dataset represents all anomalous graphs in the total dataset. The example of visualization graph for three domain datasets is shown in Fig. 3 and the detailed information of three types of graph anomaly dataset is as follows:

**Table 1:** Statistics and properties of graph anomaly datasets.

Datasets	BZR	DHFR	COX2	ENZYMES	IMDB	AIDS	NCI1	MMP	HSE	p53
Nodes	35.75	42.43	41.22	32.63	19.77	15.69	29.87	17.62	16.89	17.92
Edges	38.36	44.54	43.45	62.14	96.53	16.2	32.3	17.98	17.23	18.34
Graphs	405	467	467	600	1,000	2,000	4,110	7,558	8,417	8,903
Anomaly ratio	21.23%	39.02%	21.84%	16.67%	50%	20%	49.95%	17.76%	5.2%	8.74%

- *Molecule anomaly graphs*^14,46^: BZR, COX2, DHFR, NCI1, AIDS, MMP, HSE and p53 are molecule graph datasets. A graph represents a molecule. Atoms are modeled graph nodes and edges are chemical bonds including additional chemical attributes, where anomalous graphs have different semantic or structure pattern with the majority of graphs. For instances, we consider molecule graphs against HIV as anomaly because HIV is difficult to be treated and is rare for AIDS dataset. And like MMP, HSE and p53 from the Tox21 dataset^47^ which is a famous chemicals toxicity test program, anomalous graphs indicate the structure pattern of chemical compounds has the potential to adverse health effects.
- *Protein anomaly graphs*^46^: ENZYMES is a protein graph dataset where the secondary structure elements of protein is as nodes and node attribute information is described according to their types, i.e., helix and sheet, including several physical and chemical information. The edge is built according to the nodes neighbor relation based on the amino acid sequence or one of three nearest neighbors in space. The anomalous graphs are not one of the origin six classes^48^ or not reflect the catalyzed chemical reaction for the given protein graph.
- *Social network anomaly graphs*^46,49^: IMDB describes a movie collaboration relation graph and is from ego-networks of actors/actress in their played movies, where actors/actress are as nodes, and an edge is built by the possibility of them appearing in the same movie. The anomalous graphs are considered as one of action and romance genres.

## Evaluation

### Baselines

Due the little research for GLAD, we select the two-stage strategy which utilizes the shallow detectors to recognize anomalous graphs based on graph level vector representation as our comparing methods. We first select FGSD^29^ which learned graph embedding representations by a histogram of all node spectral distances as the graph representation model. Then, we use the isolation forest (IF)^30^, local outlier factor (LOF)^31^, and one-class support vector machine (OCSVM)^32^ as anomaly detectors. Thus, baselines includes FGSD-IF, FGSD-LOF and FGSD-OCSVM models. Furthermore, the latest end-to-end GLAD model GLocalKD^15^ utilized the node level and graph level random distillation to detect anomalous graphs as one of baselines.

### Parameter settings

In the GLADC experiments, graph encoder in dual-graph encoder module and graph encoder module share the same graph encoder which consists of two GCN layers, and the dimension of each layer to $d-256-128$, where *d* is the dimension size of node attribute features. The dimension set of decoder is $128-256-d$. We select the Adam^50^ method as the optimizer of GLADC with the learning rate of 0.0001 (0.00001 on MMP, HSE and p53). The batch size is 300 for all datasets apart from MMP, HSE and p53 datasets with 2000. The number of epochs is set 100 for all datasets. The $\eta$ and $\tau$ are set 1 and 0.2 respectively. For FGSD model, we reproduce the optimized results referred in^14^. For GLocalKD model, we retain the parameter setting of origin paper and report the optimized experiment results.

### Experimental implementation

To alleviate the number imbalance problem of normal and abnormal graphs, DLADC uses the normal graphs as training set to learn normal graph representations, and evaluates the error of abnormal graph representations in test set. In experiment, we use the five-fold cross-validation method to split datasets including EZR, DHFR, COX2, ENZYMES, IMDB, AIDS and NCI1, and remove the abnormal graphs of training set to train model. Similarly, we also remove the abnormal graphs of training set based on existing training and test dataset for MMP, HSE and p53, where the ratio of training set and test set is 31:1, 31:1 and 32:1, respectively. Besides, we run these datasets five times with different random seeds.

**Table 2:** AUC values (mean ± std) of graph level anomaly detection on ten datasets.

Datasets	FGSD-IF	FGSD-LOF	FGSD-OCSVM	GLocalKD	GLADC
MMP	0.311 ± 0.004	0.376 ± 0.001	0.578 ± 0.002	0.677 ± 0.004	**0.696** ± **0.042**
HSE	0.603 ± 0.008	0.608 ± 0.000	0.522 ± 0.002	0.587 ± 0.024	**0.618** ± **0.110**
p53	0.340 ± 0.007	0.416 ± 0.000	0.510 ± 0.001	0.639 ± 0.146	**0.649** ± **0.216**
BZR	0.564 ± 0.004	0.635 ± 0.000	0.598 ± 0.001	0.672 ± 0.091	**0.715** ± **0.067**
DHFR	0.475 ± 0.003	0.441 ± 0.000	0.419 ± 0.000	0.604 ± 0.029	**0.612** ± **0.041**
COX2	0.489 ± 0.005	0.607 ± 0.000	0.458 ± 0.001	0.606 ± 0.048	**0.615** ± **0.044**
ENZYMES	0.520 ± 0.004	0.555 ± 0.000	0.550 ± 0.000	0.442 ± 0.097	**0.583** ± **0.035**
IMDB	0.466 ± 0.003	0.445 ± 0.000	0.576 ± 0.001	0.538 ± 0.018	**0.656** ± **0.023**
AIDS	**0.994** ± **0.000**	0.578 ± 0.001	0.832 ± 0.000	0.990 ± 0.004	0.993 ± 0.005
NCI1	0.331 ± 0.001	0.632 ± 0.000	0.506 ± 0.000	**0.684** ± **0.015**	0.683 ± 0.011

Significant values are in bold.

### Experimental results

To test the performance of our GLADC model, we first perform anomaly detection evaluation with baselines on public graph anomaly datasets. Second, we analyze the ability of different modules from GLADC model for anomaly detection, and then explore the impact of parameters, efficiency analysis experiments and GLADC model discussion.

#### Anomaly detection performance

We conduct graph level anomaly detection experiments on ten available datasets to evaluate the efficiency of GLADC with the above four baselines. We use AUC value that is commonly used in anomaly detection analysis following in^10,15^ as anomaly detection measure metric. At the same time, we conduct experiments according to the training way mentioned and the final experimental results are summarized in Table 2.

According to comparative evaluation experimental results: (1) our model has the significant performance improvement with baselines for different types of anomaly graph datasets and has the strongly adaptive ability. Specifically, the outstanding performance is to 0.583 and 0.656 in ENZYMES and IMDB respectively compared with other baselines. For AIDS and NCI1 datasets, our results are very close to the best contenders. The main reason is that GLADC model is an end-to-end graph level anomaly detection framework, which integrates node level and graph level representations learning and anomaly detection optimization. Thus, GLADC can effectively learn normal graph representations and differ anomalous graph pattern representations. Furthermore, we can note that the two-stage methods achieve lower performance in most datasets, which demonstrates that designing an end-to-end anomaly detection framework is effective for different domain datasets.

#### Ablation studies

**Figure 4:**
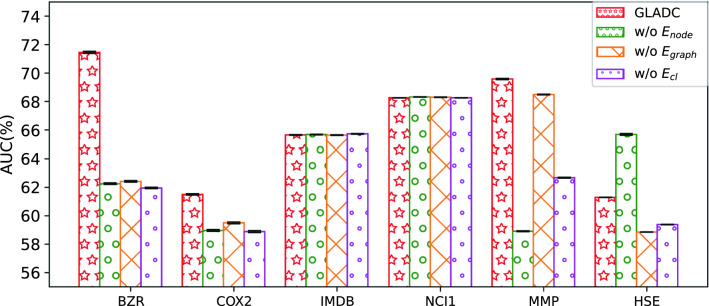
Ablation analysis for GLADC model on six datasets.

In this section, we aim to further analyze how the efficiency of different designed modules of GLADC model is. Thus, we conduct the ablation analysis experiments on six datasets as shown in Fig. 4, where $w/o E_{node}$, $w/o E_{graph}$ and $w/o E_{cl}$ indicate the corresponding components are removed from GLADC model respectively. We observe that GLADC model has the best performance among these variants apart from IMDB and NCI1 that the performance is similar with variants of GLADC. Furthermore, the performance of GLADC without graph contrastive learning is very poor on BZR, COX2, MMP and HSE, which demonstrates that the designed graph contrastive learning paradigm is effective to enhance graph representation and graph anomaly detection. Another discovery is that when we only consider global graph anomaly detection, the performance drops on COX2 and MMP but $w/o E_{node}$ gets the better performance on HSE. This shows that graph level anomaly detection needs to consider multiple strategies for different types of graph data, and our model can meet the request of anomalous graph detection for different domain data.

#### Parameter analysis

**Figure 5:**
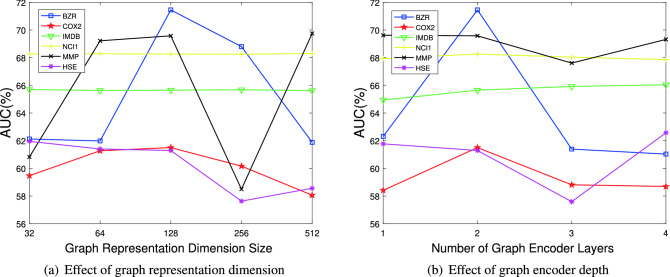
Parameter analysis for GLADC on six datasets w.r.t different values for graph representation dimension and graph encoder depth.

*Effect of representation dimension*. To explore the impact of graph representation dimension for anomalous graphs detection, we organize experiments on six datasets where the dimension is varied from 32 to 512. The performance AUC values are reported in Fig. 5a. We observe that GLADC model keeps a stable performance on most datasets apart from BZR, HSE and MMP that the performance of dimension as 256 drops dramatically. Besides, the best graph representation dimension is 128 or 256 for most datasets. The reason is that too high dimension will influence graph representation error evaluation.

*Effect of graph encoder depth* To examine the influence of the depth of graph encoder in our GLADC model on the six datasets, the number of graph encoder layer is varied from 1 to 4. As shown in Fig. 5b, when the graph encoder layers are 2, the experimental results of GLADC on BZR, COX2, NCI1, MMP and HSE are the best, and the best layer of GLADC is 3 on IMDB. This also reflects that more layers may cause the over smoothing of graph representation, resulting in the dropping of the performance of GLADC.

#### Efficiency analysis

**Figure 6:**
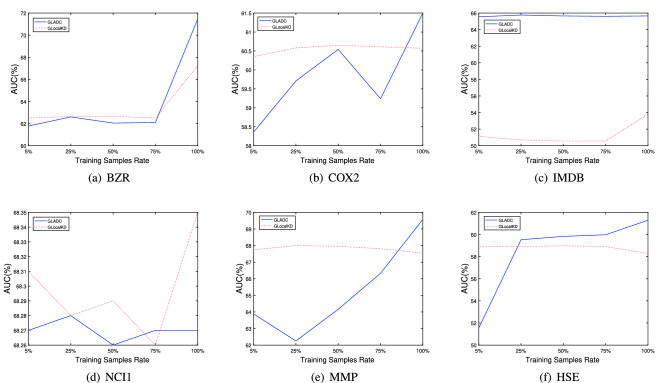
The efficiency analysis of GLADC for different training sample rates on six datasets compared with GLocalKD model.

To explore the performance of GLADC model on different training sample rates, we conduct efficiency analysis with GLocalKD method on six datasets as shown in Fig. 6. Specifically, we vary the training sample rates from 0.05 to 1 and the test dataset keeps origin setting. We have the following observations: first, GLADC outperforms GLocalKD method for different training rates on all datasets apart from NCI1. Second, GLADC model also keeps the competitive performance in low training rates on IMDB and NCI1. This is benefited from the reconstruction way and graph contrastive learning paradigm which enhances graph representations in node level and graph level and captures the graph anomaly pattern. Besides, though GLADC does not outperform GLocalKD model on NCI1, the result values are very close. These also reflect proposed GLADC model can equip with great efficiency in large graph set for GLAD task.

#### Discussion

In the section, we discuss two interesting problems about how is the performance of training set containing different numbers of anomalous graphs and how is the performance of test set with small ratio of anomalous graphs for proposed GLADC model. In order to analyze experimental results of the different numbers of anomalous graphs in the training set, we recycle some graph anomalies from removed anomalous graphs in the training set. We also decrease the number of graph anomalies in the test set to verify the efficiency of GLADC when the normal graphs are trained in the training set. Tables 3 and 4 report the experimental results, where “–” means not available.

We can observe that when some anomalous graphs are recycled on the training set, the performance of all datasets becomes lower compared with no adding anomalies except on the IMDB and NCI1, where the AUC values keep stable. The reason is that the number of normal and abnormal graphs on the test set is similar on the two datasets. As shown in Table 4, when we decrease the number of anomalous graphs in test set, the experimental results start to drop on BZR, COX2 and IMDB, but the performance is better than more anomalous graphs on test set for NCI1 and MMP, which shows the proposed GLADC model is reasonable and effective for different types of graph anomaly datasets.

**Table 3:** AUC values (mean ± std) of different numbers of anomalous graphs in training set.

Number	BZR	COX2	IMDB	NCI1	MMP	HSE
10	0.619 ± 0.049	0.583 ± 0.061	0.657 ± 0.023	0.683 ± 0.011	0.679 ± 0.042	0.590 ± 0.264
50	0.619 ± 0.049	0.584 ± 0.061	0.656 ± 0.023	0.683 ± 0.011	0.676 ± 0.068	0.590 ± 0.160
100	–	–	0.657 ± 0.023	0.683 ± 0.011	0.678 ± 0.102	0.586 ± 0.201
200	–	–	0.656 ± 0.023	0.683 ± 0.011	0.679 ± 0.076	0.587 ± 0.095
500	–	–	–	0.683 ± 0.011	0.678 ± 0.081	0.589 ± 0.105
No	0.715 ± 0.067	0.615 ± 0.044	0.656 ± 0.023	0.683 ± 0.011	0.696 ± 0.042	0.618 ± 0.110

**Table 4:** AUC values (mean ± std) of small number of anomalous graphs in test set, where () indicates the existing number of graph anomalies in the original test set.

Number	BZR(17)	COX2(21)	IMDB(100)	NCI1(411)	MMP(200)	HSE(10)
10	0.620 ± 0.044	0.548 ± 0.125	0.550 ± 0.087	0.767 ± 0.052	0.740 ± 0.137	0.618 ± 0.110
50	–	–	0.519 ± 0.061	0.656 ± 0.010	0.638 ± 0.093	0.570 ± 0.164
Original	0.715 ± 0.067	0.615 ± 0.044	0.656 ± 0.023	0.683 ± 0.011	0.696 ± 0.042	0.618 ± 0.110

## Conclusion

This work aims to explore the problem of graph level anomaly detection with little attention. The proposed GLADC first tries to introduce contrastive learning method to improve the performance of graph level anomaly detection. We design an end-to-end and effective graph level anomaly evaluation paradigm, which utilizes contrastive learning strategy to enhance node level and graph level representations for local and global graph anomalies detection. Through the inference of latent graph representations from special training way, we can differ anomalous graphs from given graph set. Furthermore, extensive experiments including ablation study, efficiency analysis and model discussion demonstrate the outstanding performance of proposed GLADC. It is noted that the performance of GLADC framework on most molecular graph anomaly datasets achieves great success, which can be further explored in drug discovery research. In the future work, we will continue to extend our method for other research directions.

## Data Availability

The datasets used in this work are available at https://chrsmrrs.github.io/datasets/.

## Accession codes

The code of this work is available at https://github.com/XuexiongLuoMQ/GLADC.

## References

[1] Ma X (2021). A comprehensive survey on graph anomaly detection with deep learning. IEEE Trans. Knowl. Data Eng..

[2] Song Z, Yang X, Xu Z, King I (2022). Graph-based semi-supervised learning: A comprehensive review. IEEE Trans. Neural Netw. Learn. Syst..

[3] Aggarwal CC, Wang H (2010). Graph data management and mining: A survey of algorithms and applications. Managing and Mining Graph Data.

[4] Borgwardt KM (2005). Protein function prediction via graph kernels. Bioinformatics.

[5] Zhao, X. *et al.* Deep reinforcement learning guided graph neural networks for brain network analysis. arXiv preprint arXiv:2203.10093 (2022). 10.1016/j.neunet.2022.06.035 35853320

[6] Lagraa S (2021). A simple graph embedding for anomaly detection in a stream of heterogeneous labeled graphs. Pattern Recognit..

[7] Luo, X. *et al.* Comga: Community-aware attributed graph anomaly detection. In *WSDM*, 657–665 (2022).

[8] Jin, M. *et al.* Anemone: Graph anomaly detection with multi-scale contrastive learning. In *CIKM*, 3122–3126 (2021).

[9] Ding, K., Li, J., Agarwal, N., & Liu, H. Inductive anomaly detection on attributed networks. In *IJCAI*, 1288–1294 (2021).

[10] Liu Y (2021). Anomaly detection on attributed networks via contrastive self-supervised learning. IEEE Trans. Neural Netw. Learn. Syst..

[11] Liu, C. *et al.* Graph pooling for graph neural networks: Progress, challenges, and opportunities. arXiv preprint arXiv:2204.07321 (2022).

[12] Ding, K., Li, J., Bhanushali, R., & Liu, H. Deep anomaly detection on attributed networks. In *SDM*, 594–602 (2019).

[13] Fan, H., Zhang, F., & Li, Z. Anomalydae: Dual autoencoder for anomaly detection on attributed networks. In *ICASSP*, 5685–5689 (2020).

[14] Zhao L, Akoglu L (2021). On using classification datasets to evaluate graph outlier detection: Peculiar observations and new insights. Big Data.

[15] Ma, R., Pang, G., Chen, L., & van den Hengel, A. Deep graph-level anomaly detection by glocal knowledge distillation. In *WSDM*, 704–714 (2022).

[16] Müller, E., Sánchez, P. I., Mülle, Y., & Böhm, K. Ranking outlier nodes in subspaces of attributed graphs. In *ICDE Workshops*, 216–222 (2013).

[17] Perozzi, B., & Akoglu, L. Scalable anomaly ranking of attributed neighborhoods. In *SDM*, 207–215 (2016).

[18] Sanchez, P. I., Muller, E., Laforet, F., Keller, F., & Bohm, K. Statistical selection of congruent subspaces for mining attributed graphs. In *ICDM*, 647–656 (2013).

[19] Sánchez, P. I., Müller, E., Irmler, O., & Böhm, K. Local context selection for outlier ranking in graphs with multiple numeric node attributes. In *SSDBM*, 16:1–16:12 (2014).

[20] Perozzi, B., Akoglu, L., Sánchez, P. I., & Müller, E. Focused clustering and outlier detection in large attributed graphs. In *KDD*, 1346–1355 (2014).

[21] Wu Z (2020). A comprehensive survey on graph neural networks. IEEE Trans. Neural Netw. Learn. Syst..

[22] Zheng, Y. *et al.* From unsupervised to few-shot graph anomaly detection: A multi-scale contrastive learning approach. arXiv preprint arXiv:2202.05525 (2022).

[23] Zhang, J., Wang, S., & Chen, S. Reconstruction enhanced multi-view contrastive learning for anomaly detection on attributed networks. arXiv preprint arXiv:2205.04816 (2022).

[24] Ding, K., Zhou, Q., Tong, H., & Liu, H. Few-shot network anomaly detection via cross-network meta-learning. In *WWW*, 2448–2456 (2021).

[25] Nguyen, H. T., Liang, P. J., & Akoglu, L. Anomaly detection in large labeled multi-graph databases. arXiv preprint arXiv:2010.03600 (2020).

[26] Shervashidze N, Schweitzer P, Van Leeuwen EJ, Mehlhorn K, Borgwardt KM (2011). Weisfeiler-lehman graph kernels. J. Mach. Learn. Res..

[27] Neumann M, Garnett R, Bauckhage C, Kersting K (2016). Propagation kernels: Efficient graph kernels from propagated information. Mach. Learn..

[28] Narayanan, A. *et al.* graph2vec: Learning distributed representations of graphs. arXiv preprint arXiv:1707.05005 (2017).

[29] Verma, S., & Zhang, Z.-L. Hunt for the unique, stable, sparse and fast feature learning on graphs. In *Advances in Neural Information Processing Systems*, vol. 30 (2017).

[30] Fei, T. L., Kai, M. T., & Zhou, Z. H. Isolation forest. In *ICDM* (2008).

[31] Breunig, M. M., Kriegel, H.-P., Ng, R. T., & Sander, J. Lof: Identifying density-based local outliers. In *SIGMOD*, 93–104 (2000).

[32] Schölkopf, B., Williamson, R. C., Smola, A., Shawe-Taylor, J., & Platt, J. Support vector method for novelty detection. In *Advances in Neural Information Processing Systems*, vol. 12 (1999).

[33] Chen, T., Kornblith, S., Norouzi, M., & Hinton, G. A simple framework for contrastive learning of visual representations. In *ICML*, 1597–1607 (PMLR, 2020).

[34] He, K., Fan, H., Wu, Y., Xie, S., & Girshick, R. Momentum contrast for unsupervised visual representation learning. In *CVPR*, 9729–9738 (2020).

[35] Wang Y, Wang J, Cao Z, Barati Farimani A (2022). Molecular contrastive learning of representations via graph neural networks. Nat. Mach. Intell..

[36] Veličković, P. *et al.* Deep graph infomax. arXiv preprint arXiv:1809.10341 (2018).

[37] Peng, Z. *et al.* Graph representation learning via graphical mutual information maximization. In *WWW*, 259–270 (2020).

[38] Zhu, Y. *et al.* Graph contrastive learning with adaptive augmentation. In *WWW*, 2069–2080 (2021).

[39] You Y (2020). Graph contrastive learning with augmentations. Adv. Neural Inf. Process. Syst..

[40] Sun, M., Xing, J., Wang, H., Chen, B., & Zhou, J. Mocl: Contrastive learning on molecular graphs with multi-level domain knowledge. arXiv preprint arXiv:2106.04509 (2021). 10.1145/3447548.3467186 PMC9105980 35571558

[41] Jin, M. *et al.* Multi-scale contrastive siamese networks for self-supervised graph representation learning. arXiv preprint arXiv:2105.05682 (2021).

[42] Xia, J., Wu, L., Chen, J., Hu, B., & Li, S. Z. Simgrace: A simple framework for graph contrastive learning without data augmentation. In *WWW*, 1070–1079 (2022).

[43] Kipf, T. N., & Welling, M. Semi-supervised classification with graph convolutional networks. In *ICLR* (2017).

[44] Van den Oord, A., Li, Y., & Vinyals, O. Representation learning with contrastive predictive coding. arXiv e-prints arXiv:1807.03748 (2018).

[45] Berman HM (2000). The protein data bank. Nucleic Acids Res..

[46] Morris, C. *et al.* Tudataset: A collection of benchmark datasets for learning with graphs. arXiv preprint arXiv:2007.08663 (2020).

[47] Kersting, K., Kriege, N. M., Morris, C., Mutzel, P., & Neumann, M. Benchmark data sets for graph kernels (2016).

[48] Schomburg I (2004). Brenda, the enzyme database: Updates and major new developments. Nucleic Acids Res..

[49] Xie, H., Ma, J., Xiong, L. & Yang, C. Federated graph classification over non-IID graphs. In *Advances in Neural Information Processing Systems*, vol. 34 (2021).

[50] Kingma, D. P., & Ba, J. Adam: A method for stochastic optimization. arXiv preprint arXiv:1412.6980 (2014).

